# Fitness and psychological effects of tele-exercise in healthy populations. Preliminary study

**DOI:** 10.3389/fdgth.2024.1496196

**Published:** 2024-11-12

**Authors:** Alberto Fucarino, Giovanna Zimatore, Antonio Fabbrizio, Nuno D. Garrido, Victor Machado Reis, José Vilaça-Alves, Martina Sausa, Barbara Matteo, Rafael Peixoto, Paulina Perušina, Aleksandra Aristova, Andrea De Giorgio, Carlo Baldari, Filippo Macaluso, Enzo Iuliano, Manuela Cantoia

**Affiliations:** ^1^ Department of Theoretical and Applied Sciences, eCampus University, Novedrate, Italy; ^2^ Research Center in Sports Sciences, Health Sciences and Human Development, CIDESD, Vila Real, Portugal; ^3^ Sport Sciences Departments, University of Trás-os-Montes e Alto Douro, Vila Real, Portugal; ^4^ Rijeka Sports Association for Persons with Disabilities, Rijeka, Croatia; ^5^ Latvian Sports Federations Council, Riga, Latvia; ^6^ Department of Biomedicine, Neurosciences and Advanced Diagnostics, University of Palermo, Palermo, Italy

**Keywords:** tele-exercise, physical fitness, psychological well-being, healthy populations, lifestyle behaviors

## Abstract

**Background:**

The study investigates the impact of tele-exercise on physical fitness and psychological well-being in healthy individuals. Tele-exercise, facilitated by technology, offers a flexible and accessible alternative to traditional exercise, particularly beneficial during restricted in-person interactions.

**Methods:**

In this study, 52 participants were divided into three groups: athletes, women, and young adults. They took part in an eight-week tele-exercise program, either synchronously or asynchronously. Physical fitness was evaluated using tests such as the 2-Minute Step and Curl Up Test, while psychological well-being was assessed using the Psychological General Well-Being Index (PGWBI) and Perceived Stress Scale (PSS-10).

**Results:**

Significant improvements in physical fitness and psychological well-being were observed in post-intervention across all groups, regardless of training mode. In the fitness tests, a significant improvement was obtained in the 2-Minute-Step (*p* = 0.004), in the curls up (*p* = 0.017), and in squats test (*p* = 0.004). In the forward bending test, the increment was very close to the significance (*p* = 0.051). In the psychological well-being tests, both PGWBI and WHO-5 scores increased after the training (*p* = 0.024 and *p* = 0.001 respectively) with no significant change in the PSS-10 score. The study found that tele-exercise can effectively introduce physical activity to previously inactive individuals and motivate them to adopt healthier lifestyle behaviors.

**Conclusions:**

The TELEexe4ALL project demonstrates the potential of tele-exercise to improve physical fitness and psychological well-being. The study suggests that tele-exercise is a feasible and well-accepted approach for enhancing overall wellness in healthy populations.

## 1 Introduction

Technological progress has brought about a significant transformation in various aspects of our lives, including the healthcare sector (1). One noteworthy advancement in this domain is the advent of tele-exercise, which involves utilizing technologies to remotely administer exercise-based interventions. Thus, tele-exercise allows for exercise programs to be delivered without the need for physical proximity, simultaneously expanding access to healthcare services (2). This approach has garnered growing interest and acknowledgement as a pioneering method to encourage physical activity and enhance health results, especially in circumstances where in-person interactions are restricted or unattainable (3–5). Hence, tele-exercise has traditionally been utilized as a means of rehabilitation, as one key benefit is its inherent flexibility and convenience since it eliminates the need for individuals to physically reach exercise facilities or clinics. As a result, it is an easily accessible option for those with mobility impairments or those living in distant locales. Remote training delivery has shown promising potential in different populations, ranging from people with chronic condition (6–8), such as Parkinson (9) or Diabetes (10), to people in rehabilitation after a stroke (11), a spinal cord injury (12) or cardiac problems (13), to healthy ones, seeking personalized exercise programs such as elders (14–17).

The literature has also shown that exercise is able to improve both the cognitive (18, 19) and emotional sphere (16, 20, 21), benefits that also seem to be maintained by tele-exercise, although the research in this regard in healthy people is still scarce (22). To all aforementioned benefits should be added the fact that tele-exercise allows both personalized prescriptions and real-time monitoring through smart devices (23), by giving the opportunity to the trainers to tailor interventions/training based on individual needs and monitor progress more effectively. However, considering tele-exercise as an exclusive mode of physical rehabilitation would be wrong and limiting. There has been a steady rise in the number of applications designed for the wellness of the general population (24–27). Additionally, the scientific community is actively researching the advantages of tele-exercise in enhancing the overall quality of life and habits of those subjects that are already in good health (16, 28, 29) and choose to stay active through remote means. Tele-exercise can benefit people of all ages and backgrounds, including athletes, seniors, and youth. Tele-exercise's positive impact on both physical wellness and mental well-being is universal, making it an effective method for enhancing overall health and well-being (14, 16, 22, 30–33).

Despite the growing evidence supporting the benefits of tele-exercise, several challenges and considerations should be addressed to ensure its successful implementation, including technological infrastructure, privacy and security concerns, and the need for appropriate training and support for both trainers and participants. Further, to our best knowledge, the effects on tele-exercise on the psychological sphere of healthy people have not been investigated. With the purpose of support and stimulate exercise, improve physical wellness and psychological wellbeing, ensuring privacy and security, it has been designed the European project called “TELEexe4ALL”, characterized—as far as we know for the first time—by designing a dedicated platform that can be accessed by people through synchronous and asynchronous mode. The open-source platform TELEexe4ALL (34) provides tele-exercise sessions based on end-user characteristics, creating homogeneous and inclusive classes. Through this platform we wanted to investigate the effects of synchronous and asynchronous tele-exercise in a heterogenous group of healthy people (from sedentary subjects to athletes) by investigating their state of well-being and physical activity. Here we present preliminary data, discussing possible future developments and scenarios.

## 2 Materials and methods

### 2.1 Participants

A total of 52 participants (34 females and 18 males, aged 30.65 ± 8.86 years) were recruited for the present preliminary study and clustered in 3 intervention groups: 16 participants were athletes of both gender (ATHLETES group), 17 participants were adult women (WOMEN group), and 19 participants were young adults of both genders (YOUNG group). Each participant had the opportunity to follow a tele-exercise course either in asynchronous (*N* = 31; AS group) or synchronous mode (*N* = 21; SY group). The participants’ main characteristics and the number of participants that decided to follow the course in asynchronous or synchronous mode are shown in Table 1.

**Table 1:** Participants’ characteristics.

Group	*N*	Age (y)	Gender (M/F)	Mode (AS/SY)	W (Kg)	H (cm)	BMI (kg/m^2^)
ATHLETES	16	32.1 ± 12.1	7/9	10/6	72.2 ± 12.2	175.6 ± 9.9	23.3 ± 2.4
WOMEN	17	36.4 ± 3.9	0/17	13/4	60.9 ± 10.0	164.2 ± 5.8	22.6 ± 3.2
YOUNG	19	23.9 ± 2.3	11/8	8/11	69.9 ± 11.9	172.6 ± 6.3	23.4 ± 3.4
All	52	30.6 ± 8.9	18/34	31/21	67.7 ± 12.2	170.7 ± 8.8	23.1 ± 3.0

Age, Weight and Height are reported as means ± standard deviation, Gender and Mode are re-ported as numerosity.

AS, asynchronous; SY, synchronous.

Only one participant has a BMI > 30 (32.8) kg/m^2^.

### 2.2 Enrollment

The enrollment into the three intervention groups was performed using a convenience sampling based on the inclusion and exclusion criteria reported in Table 2. The study was promoted using the TELEexe4ALL open-source platform, the social media channels of the project (Twitter, Facebook, Instagram), the social channels of the TELEexe4ALL partners, with the aim of recruiting potential participants. Subsequently, each potential participant filled in a questionnaire on personal data to assess the inclusion and exclusion criteria. Finally, they were assigned to the respective group (ATHLETES, YOUNG, WOMEN).

**Table 2:** Inclusion and exclusion criteria.

	Inclusion criteria	Exclusion criteria
ATHLETES	• Both genders • Athlete competing at national level and registered with a sports federation from at least 3 years • Valid competitive medical certificate	• Presence of pathology, disability or other condition incompatible with the procedures proposed in this preliminary investigation • Use of drugs or medication, or other situations which could introduce a bias into the procedures of the investigation
WOMEN	• Female gender • Age between 30 and 55 years • Sedentary or normally active lifestyle • Valid non-competitive medical certificate	
YOUNG	• Both genders • Age between 18 and 29 years • Sedentary or normally active lifestyle • Valid non-competitive medical certificate	

### 2.3 Procedure

The duration of the intervention was 8 weeks. During this period the participants of WOMEN and YOUNG groups had to attend 24 exercise lessons (3 lessons/week) in telematic mode lasting 1 h per lesson. ATHLETES integrated their routine training following 16 exercise lessons (2 lessons/week). The lessons were delivered on the TELEexe4ALL open-source platform (accessible at the website www.teleexe4all.eu). Once registered, participants could choose whether to attend the lessons in synchronous or asynchronous mode and had access to all the video-lessons scheduled for their respective groups.

#### 2.3.1 Synchronous mode

In this mode, all the lessons were conducted by an instructor live and in real time. Participants had to perform a series of exercises shown via video-lessons, while an experienced instructor monitored their execution and corrected them if necessary. In the synchronous mode, participants had the opportunity to interact with instructors and other participants in real-time using the meeting functionality of the TELEexe4ALL platform. The exercises shown in all the video-lessons were accurately demonstrated by an expert instructor that showed the correct execution to be performed and tried to describe the most common mistakes to be avoided. Furthermore, all the exercises proposed could be performed without the aid of any gym tool or machinery as they were designed to be feasible in a home environment.

During the lessons, the ratio between the supervising instructor and the participants ranged from 1/2 to 1/8.

#### 2.3.2 Asynchronous mode

In this mode, all the lessons were administered via pre-recorded video-lessons. Consequently, in the asynchronous mode, no real-time supervision by expert instructors was provided. At any time, participants had access to the same video-lessons used for the synchronous lessons, and they simply had to reproduce the exercises autonomously. Participants were asked to carry out the 24 video-lessons of training with a frequency of 3 video-lessons per week, on non-consecutive days. The platform tracked participants’ access to the video-lessons to evaluate actual compliance with scheduled days and to avoid over- or undertraining.

As in the synchronous mode, all the exercises could be performed without the aid of any gym tool or machinery.

The type of training varied based on the specific needs of the target population. The ATHLETES group performed a training protocol focusing on different strength and conditioning exercises designed for prevention of injury. The exercises aimed to produce a conditioning of the major muscles’ groups and joints. The intensity of the workouts was initially 4 RPE points (rate of perceived effort), up to a maximum of 6 RPE using Borg's CR10 scale (35). The WOMEN group performed a functional training consisting of mixed workouts to improve both cardiovascular and muscular fitness, with a particular focus on the major muscles’ groups to maximize the health benefit induced by training. The intensity of the workouts was initially 4 RPE points, up to a maximum of 8 points based on participants’ fitness level. The YOUNG group performed mixed workouts with the aim of improving cardiovascular fitness, muscular fitness, but also coordination skills. This training was designed to promote an overall improvement of all motor skills. Also in this case, the intensity of the workouts will initially be 4 RPE points, up to a maximum of 8 points depending on participants’ fitness level.

At the beginning and at the end of the 8 weeks of intervention, a series of tests were performed to evaluate the differences between pre- and post-intervention scores as explained in the following section. The tests were both physical (fitness tests) and psychological (psychological well-being, perceived stress). In the post-intervention assessment, a qualitative survey was carried out to the participants to have feedback concerning their opinions on the activity performed.

### 2.4 Measures

#### 2.4.1 Fitness tests

The fitness tests were selected as they do not require any equipment and specific skills, and they could be easily and safely administered in a domestic environment. The tests were performed in an autonomous manner by the participants of the WOMEN and YOUNG groups (*N* = 35); the participants of the ATHLETES group did not perform any fitness tests since their physical fitness level was not comparable with amateur trainees. The tests were administered at baseline and at the end of the intervention to evaluate any significant changes in these scores due to training.

The following tests were used:

2-min step test: It is a field test used to assess aerobic fitness. In this test, the participant must stand next to the wall and mark on the wall the corresponding point halfway between the kneecap and the iliac crest. Then, the participant marches in place for two minutes, raising his knees to the point that he/she marked on the wall and must count the number of steps he/she can take in two minutes. Resting is allowed during the 2 min of the test (36).
Curl up test: This test is a field test aiming to assess the strength and endurance of the abdominal muscles. The participant must lie down on a mat with their knees bent at 90°, their feet flat on the floor and their hands resting on their thighs. Then, the participants must perform a series of carl ups sliding their hands up their thighs until the fingertips touch the top of the kneecaps and then return to the starting position. The two movements (carl up and returning to the starting position) must be performed in approximately 3 s. To maintain the rhythm during the test it is advisable to use a metronome set to 20 beats per minute. The athlete must count the number of curls he/she can perform as long as he/she can maintain the rhythm of the metronome (37).
Forward bending test: This Standing test was adapted from the “Sit and reach test” (38, 39) to determine the flexibility of the spine and hips. The participant in standing position, with his/her knees extended, must bend forward trying to touch the lowest point of the lower limbs with his/her fingertips. A score is assigned based on the following criteria: 1 point is awarded if the participant reaches the thighs with his/her fingertips, 2 points if he/she reaches the knees, 3 points if he/she reaches the shins, 4 points if he/she reaches the ankles, 5 points if he/she touches the feet, and 6 points if he/she touches the floor.
Squat test: This field test aims to assess the strength and endurance of the lower limb muscles. The participants must stand approximately 40–50 cm in front of a chair, with the back facing the chair and with the feet shoulder-width apart. Then, the participant must squat down until he/she lightly touches the chair with his/her buttocks (but without sitting down), and then stand back up and repeat this sequence of movements until they are unable to continue. The participant must count the total number of squats performed. Resting is not allowed and the two movements (squat down and return to the starting position) must be performed in approximately 3 s (40).

#### 2.4.2 Psychological tests

As for Fitness tests, the tests psychological tests were administered at baseline and at the end of the intervention to evaluate any significant changes in these scores due to training. All the tests were administered via TELEexe4ALL platform. The following psychological tests were administered to all participants to assess the influence of tele-exercise on psychological well-being:

• Perceived stress scale (PSS-10): We used the Italian version of the Perceived Stress Scale (41, 42), which is a10-item self-report measure of perceived stress to evaluate the degree to which respondents appraise events as stressful during the past month. Items are rated on a 5-point Likert scale (from 0 = never, to 4 = very often), and higher total scores indicate greater perceived stress.
• World health organization—five well-being index (WHO-5): It is a questionnaire that is commonly used to assess subjective well-being and mental health (43). It is a self-reported measure that consists of five items, each focusing on different aspects of well-being. Participants are asked to rate each item on a 6-point Likert scale ranging from 0 to 5, with higher scores indicating greater well-being. The items assess feelings of happiness, interest in daily activities, energy levels, and overall satisfaction with life.
• Short form of the psychological general well-being index (PGWBI a/b): The questionnaire assesses both individual's subjective well-being and psychological health. The Italian version (44) is a short self-report measure that consists of 6 items, each one covering one dimension of well-being: anxiety, depressed mood, positive well-being, self-control, general health, and vitality. It is designed to capture both positive and negative aspects of well-being in the past month, providing a comprehensive assessment of an individual's psychological state. Each item is rated on a 6-point Likert scale, ranging from 0 (none of the time) to 5 (all of the time).

#### 2.4.3 Qualitative post survey

Three months into the conclusion of the training period, participants were contacted for a follow-up survey. Thirty-five participants (26 females; 9 males) filled in the questionnaires. The participants represented the three groups whose characteristics are described in Table 3. As for the psychological well-being tests, this survey was administered via TELEexe4ALL platform.

**Table 3:** Characteristics of the sample who completed the qualitative post survey.

Group	N	Age (y)	Gender (M/F)	Mode (AS/SY)	Weight (Kg)	Height (cm)
ATHLETES	10	32.5 ± 10.5	5/5	6/4	77.2 ± 12.7	175.6 ± 9.9
WOMEN	16	37.6 ± 6.2	0/16	12/4	61.3 ± 12.8	180.0 ± 9.7
YOUNG	9	25.5 ± 4.8	4/5	8/1	69.3 ± 9.7	163.8 ± 6.5
All	35	32.7 ± 10.6	9/26	26/9	68.0 ± 13.6	175.4 ± 8.8

Age, Weight and Height are reported as means ± standard deviation, Gender and Mode are reported as numerosity.

AS, asynchronous; SY, synchronous.

The qualitative post survey consisted of 19 items assessing: (1) the participants’ degree of satisfaction, (2) their possible increased involvement in weekly exercise after participation in the project, and (3) any suggestions or criticisms. Two items were categorical ordered items, respectively investigating the engagement in physical exercise programs before and after the participation in the present project, 14 items were 5-point Likert scale (Strongly disagree, Disagree, Neither agree nor disagree, Agree, Strongly agree), investigating the opinion of the participants on different aspects of the project (45), while the last 3 items were open-ended items, asking for eventual suggestion or criticism.

### 2.5 Statistical analysis

*A priori* power analysis indicated that a total sample size of 30 subjects was required to detect a medium effect size (f = 0.25) given a coefficient of correlation *p* = 0.60 with 80% power and alfa = 0.05, using ANOVA repeated measure, within factors.

A Kolmogorov–Smirnov Test of Normality was performed to confirm if variables were normally distributed.

#### 2.5.1 Statistical analysis of the fitness and psychological tests

Repeated measures analysis of variance (RM-ANOVA) was used to assess main time effects of methods (pre vs. post), the interaction time*groups (ATHLETES, WOMEN, YOUNG) and interaction effects, time*mode (AS, SY). All the scores obtained by the fitness and psychological tests were considered as dependent variables for the analyses. Partial Eta square (*η*^2^*p*) was also computed as an indicator of the effect size of the analysis.

Statistical analysis of qualitative post survey: Firstly, a descriptive frequency analysis performed on the two categorical items respectively investigating the engagement in physical exercises programs before and after the participation were performed. Then, another descriptive analysis (mean score ± SD) of the 14 Likert scaled items was calculated. This descriptive analysis was performed for all participants as a single group, and separately for each one of the three groups ATHLETES, WOMEN, and YOUNG. The non-parametric Kruskal-Wallis's test was successively used to compare the results of the three groups to evaluate whether significant differences existed among them. Finally, the last 3 items, that were open-ended items, were analyzed in qualitative way by grouping the suggestions and indications based on their similarity.

For all the analyses, statistical significance was defined as *p* ≤ 0.05 and all results were expressed as mean ± SD for continuous variables, or percentage and numerosity for categorical variables.

All statistical analysis was performed by SPSS version 27.0 software (SPSS Inc., Chicago, IL, USA).

## 3 Results

All the participants considered in this preliminary study results completed 100% of the proposed lessons.

### 3.1 Fitness tests

RM-ANOVA results show statistically significant differences in the pre-post administrations of the tests. At the end of the training period, participants performed a mean of 166.11 ± 42.45 steps in 2-Minute-Step test, instead of the 140.28 ± 42.65 steps they reached before starting the training. These results are significantly different (F = 9.497, *η*^2^*p* = 0.213, *p* = 0.004).

At the end of the study, participants were also able to perform a mean of 40.17 ± 19.73 curls up, instead of the previous 35.69 ± 19.56 in the pre-test (F = 6.274, *η*^2^*p* = 0.152, *p* = 0.017), and a mean of 51.28 ± 38.03 squats instead of 42.86 ± 32.27 (F = 9.640, *η*^2^*p* = 0.216, *p* = 0.004). They also registered an increase in the mean number of forward bending (F = 4.093, *η*^2^*p* = 0.105, *p* = 0.051) that is very close to significance.

Furthermore, RM-ANOVA reported a significant difference when considering the interaction with groups only in the 2-Minute Step Test with a better trend for the YOUNG trainees. No significant differences were found in the interaction time*mode (synchronous vs. asynchronous mode). The complete results are reported in Table 4.

**Table 4:** Results of the fitness tests.

	2-Minute step test		Curl up test		Forward bending test		Squat test	
	Pre	Post	Pre	Post	Pre	Post	Pre	Post
All (36)	140.28 ± 42.65	166.11 ± 42.45	35.69 ± 19.56	40.17 ± 19.73	4.75 ± 1.16	4.94 ± 1.19	42.86 ± 32.27	51.28 ± 38.03
*p*-value Pre vs. Post	*p* = 0.004^a^		*p* = 0.017^a^		*p* = 0.051		*p =* 0.004^a^	
YOUNG (19)	123.05 ± 43.33	165.58 ± 54.86	24.16 ± 10.58	27.58 ± 9.64	4.21 ± 1.13	4.47 ± 1.31	24.74 ± 9.36	29.00 ± 7.49
WOMEN (17)	159.53 ± 33.48	166.71 ± 23.57	48.59 ± 19.40	54.23 ± 18.68	5.35 ± 0.86	5.47 ± 0.80	63.12 ± 36.80	76.18 ± 43.05
*p*-value time^a^groups	*p* = 0.033^a^		*p* = 0.541		*p* = 0.458		*p* = 0.106	
SY (15)	128.80 ± 31.60	162.07 ± 53.52	25.53 ± 12.07	31.47 ± 9.19	4.33 ± 1.29	4.73 ± 1.33	26.53 ± 11.06	32.27 ± 9.86
AS (21)	148.48 ± 48.12	169.00 ± 33.59	42.95 ± 20.85	46.38 ± 22.92	5.05 ± 0.97	5.09 ± 1.09	54.52 ± 37.34	64.86 ± 44.71
*p*-value time^a^mode	*p* = 0.462		*p* = 0.497		*p* = 0.070		*p* = 0.411	

^a^ Statistically significant, SY = Synchronous, AS = Asynchronous.

### 3.2 Psychological tests

Perceived stress (PSS-10) registered low levels with no significant change after the training period as shown in Table 5 (F = 3.743, *η*^2^*p* = 0.068, *p* = 0.059). The levels of stress were aligned with data from the Italian validation (42).

**Table 5:** Results of the psychological tests.

	PSS-10		PGWBI		WHO-5	
	Pre	Post	Pre	Post	Pre	Post
All (62)	18.15 ± 5.78	19.69 ± 3.65	20.00 ± 4.35	21.63 ± 3.78	14.81 ± 4.5	16.96 ± 3.03
*p*-value Pre vs. Post	*p* = 0.059		*p* = 0.024^a^		*p* = 0.001^a^	
ATHLETES (16)	17.13 ± 5.97	18.56 ± 3.31	21.19 ± 3.85	22.44 ± 3.42	16.19 ± 4.17	17.88 ± 3.12
YOUNG (19)	17.79 ± 6.08	19.89 ± 3.81	19.58 ± 4.99	21.05 ± 4.2	15.05 ± 3.92	17.21 ± 2.80
WOMEN (17)	19.53 ± 5.33	20.53 ± 3.71	19.35 ± 4.05	21.53 ± 3.69	13.24 ± 5.14	15.82 ± 3.00
*p*-value time^a^groups	*p* = 0.849		*p* = 0.864		*p* = 0.848	
SY	18.57 ± 6.18	19.52 ± 3.94	19.48 ± 4.18	21.90 ± 4.40	14.67 ± 3.51	17.48 ± 2.62
AS	17.87 ± 5.58	19.81 ± 3.50	20.35 ± 4.50	21.45 ± 3.35	14.90 ± 5.12	16.61 ± 3.27
*p*-value time^a^mode	*p* = 0.549		*p* = 0.359		*p* = 0.384	

^a^ Statistically significant.

SY, synchronous; AS, asynchronous.

The levels of wellbeing, as detected through the short form of PGWBI, were at the ≈66% at baseline. The score significantly increased after the training showing +1.63 points on average (F = 5.376, *η*^2^*p* = 0.095, *p* = 0.024).

RM-ANOVA also showed a significant amelioration in pre-post comparison for WHO-5 test on wellbeing (F = 12.343, *η*^2^*p* = 0.195, *p* = 0.001), whose mean total score increases from 14.81 ± 4.53 to 16.96 ± 3.03, remaining largely over the cut off for depression (43). The RM-ANOVA reported no differences when considering the interaction with group and mode.

### 3.3 Qualitative post survey

Results of the descriptive frequency analysis performed on the two categorical items respectively investigating the engagement in physical exercises programs before and after participation in the project are reported in Table 6. As predictable, Athletes declared to train all day or all week both before and after the project. The frequency for the YOUNG and WOMEN groups allows for a better interpretation of the effects of the program: sedentary participants decreased from 32% to 12% and physical activity during the week increased both as once a week (from 20% to 25.71%) and more days a week (from 28.57% to 40%) habit.

**Table 6:** The engagement in physical exercises programs before and after participation in the project.

		Before (Pre)—Item 1					After (Post)—Item 2				
		Never	Once a week	Every week	Every day	Total	Never	Once a week	Every week	Every day	Total
All group	N	8	7	10	10	35	3	9	14	9	35
	%	23	20	29	29	100	9	26	40	26	100
YOUNG and Women	N	8	7	7	3	25	3	9	11	2	25
	%	32	28	28	12	100	12	36	44	8	100

The mean score ± SD of the 14 Likert scaled items calculated for all the participants considered as one group, and for each one of the three groups, as well as the results of the non-parametric Kruskal-Wallis's test comparing the results of the three groups are instead reported in Table 7 score ranged from 1 (Worst) to 5 (Best).

**Table 7:** Positive and negative assessment of the program.

Items		All	Athletes	Women	Young
3	Satisfaction	4.51 ± 0.51	4.70 ± 0.48	4.44 ± 0.53	4.44 ± 0.51
4	Motivation to be engaged in more physical activities	4.23 ± 0.81	4.50 ± 0.71	4.11 ± 0.93	4.13 ± 0.81
5	To exercise from home	3.94 ± 1.03	4.30 ± 0.82	3.89 ± 1.17	3.75 ± 1.06
6	A free of charge course	4.06 ± 0.97	4.60 ± 0.70	4.11 ± 0.93	3.69 ± 1.01
7	Instructors’ professionalism	4.29 ± 0.89	4.60 ± 0.70	4.00 ± 0.87	4.25 ± 1.00
8	The type of exercises	4.00 ± 0.91	4.20 ± 0.79	3.78 ± 0.97	4.00 ± 0.97
9	Choice between synchronous and asynchronous mode	4.03 ± 0.95	4.40 ± 0.84	4.00 ± 0.87	3.81 ± 1.05
10	Social messages for a healthy lifestyle on the platform	3.34 ± 0.97	3.50 ± 0.85	3.67 ± 0.87	3.06 ± 1.06
11	Choice among multiple time slots	4.03 ± 1.10	4.10 ± 1.37	4.11 ± 0.93	3.94 ± 1.06
12	How limiting was the lack of direct support for you?	2.09 ± 1.04	2.40 ± 1.35	1.89 ± 1.05	2.00 ± 0.82
13	Lack of contact with other participants	2.26 ± 1.09	2.80 ± 1.32	1.78 ± 0.97	2.19 ± 0.91
14	Training time slots	1.60 ± 0.91	2.20 ± 1.03^a^	1.44 ± 1.01	1.31 ± 0.60
15	Negative impact of physical activities	1.51 ± 0.92	1.80 ± 1.23	1.56 ± 1.01	1.31 ± 0.60
16	Post session overall opinion	2.03 ± 1.04	2.10 ± 1.10	2.00 ± 1.00	2.00 ± 1.10

^a^ Pairwise comparisons indicated significant differences between ATHLETES vs. WOMEN and YOUNG scores (respectively with *p* = 0.026 and *p* = 0.009).

Participants were all very satisfied with the program (score: 4.5 ± 0.51) and felt motivated to engage in more physical activities (score: 4.23 ± 0.81) with no differences among the groups (except for Training Time Slots *p* < 0.006). The most important features of the project were all scored above 3.30 on a Likert scale of 5 points. The most appreciated item was the instructors’ professionalism (4.29 ± 0.89), while the least appreciated was receiving social messages for a healthy lifestyle on the platform (3.34 ± 0.97).

The Kruskal-Wallis's test showed significant differences only in the item n. 14 “Training time slots” with a significant overall difference among the three groups (H = 10.351, *η*^2^ = 0.261, *p* = 0.006). Pairwise comparisons showed significant differences between ATHLETES vs. WOMEN and YOUNG scores (respectively with *p* = 0.026 and *p* = 0.009).

## 4 Discussion

The main objective of this study was to evaluate whether the implementation of a tele-exercise platform could benefit on a physical and psychological level different categories of users in good health, in “the post-covid era”. The technological advancements and availability of digital interconnection technologies have made exercise activities through telematics more enjoyable. Although the concept of tele-exercise dates back to the early 2000s (14), it only gained popularity a decade later (46, 47). The unfortunate period of the COVID-19 pandemic has renewed interest in physical activity carried out in a telematics mode (4, 48, 49). Tele-exercise is now a beneficial tool for everyone, not just critically ill patients or those undergoing rehabilitation, as research has shown. Some recent studies addressed online applications for exercise promotion in a telematics mode (50–52). We hope that the international scientific community will continue to explore the benefits of tele-exercise, as an effective way to improve the poor physical activity habits of a consistent number of European and Western citizens.

Indeed, we know from literature that even the programs that focus on healthy individuals could have some critical shortcomings. To ensure maximum participation rates, a tele-exercise program should not just provide a downloadable application but also a platform that can be accessed from any device. Furthermore, to cater to diverse individuals with different needs, synchronous and asynchronous training modes should be provided simultaneously.

Finally, to optimize the results, exercise programs must be as personalized as possible. These are the exact features that the TELEexe4ALL project is aimed to improve.

Furthermore, authors have recently proposed a new method for detecting the aerobic metabolic threshold (53, 54). This method relies solely on heart-rate time series, which eliminates the need for visual inspection; in future, it can be easily implemented in applications that gather data from portable heart rate monitors and during specific tele-exercise sessions. It is implied that one can automatically determine one's level of fitness and access it personally.

In this work the participants were divided into three groups and underwent a tele-exercise program for eight weeks, with different training frequencies and modes, as explained in the previous sections.

The results of the physical and psychological tests in the post-intervention showed significant improvements compared to the pre-intervention scores, regardless of the mode of training (synchronous vs. asynchronous).

In the fitness tests (that were underwent only by YOUNG and WOMEN groups), the RM-ANOVA results indicated statistically significant differences in the participants’ pre-post performances of 2-Minute Step Test, curls up, squat, and forward bending. Participation in the training program by untrained and slightly trained (young and women) allowed an increase in their fitness level after a relatively short period, regardless of age, gender, and physical test considered. In particular, analyses indicated a significant interaction time*group in the performance of the 2-Minute Step Test: YOUNG participants improved more than WOMEN, probably due to their lower level in the baseline. We know from literature that less trained individuals increase their performance higher than normally trained (see Table 4) (55). The improvement in the physical fitness outcomes obtained in the present study is in accordance with previous study and review in literature (14, 56). However, it should be noted that a comparison of the present results with the literature is complicated given that the studies present in the literature are very heterogeneous in terms of participants (often with pathologies), proposed training protocols (which are often related to rehabilitation training) and duration of the intervention.

Regular exercise not only improved physical fitness but also had a positive impact on overall psychological well-being, as measured by the WHO-5 and PGWBI questionnaires, with no difference related either to the participants’ characteristics, nor to the modality of the tele-exercise (synchronous vs. asynchronous).

Furthermore, the qualitative survey showed a good level of satisfaction of the participants concerning the program and a high level of motivation to engage in more physical activities. In detail, the possibility of planning the session slots resulted more important for athletes who already have a strict training schedule, the other groups preferred asynchronous mode and were enough motivated to complete their training program.

This data allows us to confirm that the first course of TELEExe4all project has in fact achieved its objective of promoting more active lifestyles. The project therefore not only proved useful from the point of view of improving physical fitness and from a psychological point of view, but also proved to be well accepted by the participants and feasible in its current version.

In conclusion, the study confirms the importance of physical activity in overall wellness and well-being; the results also suggest that tele-exercise can be an effective and well-received method for enhancing physical fitness and psychological well-being in healthy individuals, regardless of the participant group, exercise mode nor level of activity.

The primary goal of our work is to promote healthier lifestyle habits amongst participants, with a focus on sustaining these habits over time. Encouragingly, we have observed a rise in the number of individuals who have engaged in at least some physical activity throughout the week, as opposed to their previously sedentary state. We have found that tele-exercise is an effective means of introducing physical activity to those who were previously inactive and can serve as a catalyst for the adoption of healthier lifestyle behaviors.

In this paper, the results provide valuable insights into the potential and various benefits, both physically and psychologically, of tele-exercise and lay the groundwork for future research in this area. One more asset of this work is represented by multidisciplinary study design and the aim to offer a free platform for healthy people to exercise with fitness purpose in an environment scientifically controlled and with activities that do not need any particular device to be performed.

### 4.1 Limitation

This current preliminary analysis has some limitations, particularly on the number of participants. As the study is ongoing, tele-exercise sessions were still going on the platform to expand the sample of subjects who participate in the TELEexe4ALL project and to include other categories, such as elderly people (57, 58) and people with disabilities. Although these categories often face difficulties in interfacing with the latest technologies, in authors’ opinion they may be among those who benefit most from the practice of tele-exercise, as demonstrated by other studies (59–61). In general, it should be underlined that accessibility to this type of platform is in any case subordinate to a certain level of IT knowledge and this may be a limitation of the study as people with low IT knowledge may not be attracted by this type of proposal.

The self-reporting data may also result in a limitation due to the risk of reliability of the measures, nonetheless participants’ performance during the tele-exercise sessions resulted generally aligned with the pre-session evaluation and were checked and monitored by the instructors in both modes of training.

Finally, although economical for users and participants, the development of this type of platform could be expensive for the platform administrators in terms of development and management due to the need to ensure functionality and data security for participants. Therefore, the costs and benefits of using this type of platform should be evaluated in the long term (38).

## Data Availability

The raw data supporting the conclusions of this article will be made available by the authors, without undue reservation.
